# Supplementary material for: Bayesian refinement of protein structures and ensembles against SAXS data using molecular dynamics
Source: PLoS Comput Biol. 2017 Oct 18;13(10):e1005800. doi: 10.1371/journal.pcbi.1005800 (PMC5662244; doi:10.1371/journal.pcbi.1005800)
Supplement: S1 Archive — (BZ2) [file pcbi.1005800.s011.tar.bz2 › gromacs_4.62_bayesian_saxs_refi/share/html/online/g_dyecoupl.html]

g\_dyecoupl


| g\_dyecoupl Main Table of Contents |

**VERSION 4.6  
Sat 19 Jan 2013**

---

### Description

This tool extracts dye dynamics from trajectory files.
Currently, R and kappa^2 between dyes is extracted for (F)RET
simulations with assumed dipolar coupling as in the Foerster equation.
It further allows the calculation of R(t) and kappa^2(t), R and
kappa^2 histograms and averages, as well as the instantaneous FRET
efficiency E(t) for a specified Foerster radius R\_0 (switch -R0).
The input dyes have to be whole (see res and mol pbc options
in trjconv).
The dye transition dipole moment has to be defined by at least
a single atom pair, however multiple atom pairs can be provided
in the index file. The distance R is calculated on the basis of
the COMs of the given atom pairs.
The -pbcdist option calculates distances to the nearest periodic
image instead to the distance in the box. This works however only,for periodic boundaries in all 3 dimensions.
The -norm option (area-) normalizes the histograms.

### Files

| option | filename | type | description |
| --- | --- | --- | --- |
| **-f** | traj.xtc | Input | Trajectory: xtc trr trj gro g96 pdb cpt |
| **-n** | index.ndx | Input | Index file |
| **-ot** | rkappa.xvg | Output, Opt. | xvgr/xmgr file |
| **-oe** | insteff.xvg | Output, Opt. | xvgr/xmgr file |
| **-o** | rkappa.dat | Output, Opt. | Generic data file |
| **-rhist** | rhist.xvg | Output, Opt. | xvgr/xmgr file |
| **-khist** | khist.xvg | Output, Opt. | xvgr/xmgr file |

### Other options

| option | type | default | description |
| --- | --- | --- | --- |
| **-[no]h** | bool | no | Print help info and quit || **-[no]version** | bool | no | Print version info and quit || **-nice** | int | 19 | Set the nicelevel || **-b** | time | 0 | First frame (ps) to read from trajectory || **-e** | time | 0 | Last frame (ps) to read from trajectory || **-tu** | enum | ps | Time unit: fs, ps, ns, us, ms or s || **-[no]w** | bool | no | View output .xvg, .xpm, .eps and .pdb files || **-xvg** | enum | xmgrace | xvg plot formatting: xmgrace, xmgr or none || **-[no]pbcdist** | bool | no | Distance R based on PBC || **-[no]norm** | bool | no | Normalize histograms || **-bins** | int | 50 | # of histogram bins || **-R0** | real | -1 | Foerster radius including kappa^2=2/3 in nm |

---

http://www.gromacs.org  
gromacs@gromacs.org
